# Diversity of Aerobic Anoxygenic Phototrophs and Rhodopsin-Containing Bacteria in the Surface Microlayer, Water Column and Epilithic Biofilms of Lake Baikal

**DOI:** 10.3390/microorganisms9040842

**Published:** 2021-04-14

**Authors:** Agnia Dmitrievna Galachyants, Andrey Yurjevich Krasnopeev, Galina Vladimirovna Podlesnaya, Sergey Anatoljevich Potapov, Elena Viktorovna Sukhanova, Irina Vasiljevna Tikhonova, Ekaterina Andreevna Zimens, Marsel Rasimovich Kabilov, Natalia Albertovna Zhuchenko, Anna Sergeevna Gorshkova, Maria Yurjevna Suslova, Olga Ivanovna Belykh

**Affiliations:** 1 Limnological Institute Siberian Branch of the Russian Academy of Sciences, Ulan-Batorskaya 3, 664033 Irkutsk, Russia; andrewkrasnopeev@gmail.com (A.Y.K.); podlesnaya@lin.irk.ru (G.V.P.); poet1988@list.ru (S.A.P.); sukhanovalena17@gmail.com (E.V.S.); iren@lin.irk.ru (I.V.T.); ekaterinasiemens93@gmail.com (E.A.Z.); zhna@lin.irk.ru (N.A.Z.); kovadlo@lin.irk.ru (A.S.G.); msuslova1979@mail.ru (M.Y.S.); 2 Chemical Biology and Fundamental Medicine Siberian Branch of the Russian Academy of Sciences, Lavrentiev Avenue 8, 630090 Novosibirsk, Russia; kabilov@niboch.nsc.ru

**Keywords:** aerobic anoxygenic phototrophs, rhodopsin-containing bacteria, neuston, plankton, epilithon, Lake Baikal, *pufM*, rhodopsin

## Abstract

The diversity of aerobic anoxygenic phototrophs (AAPs) and rhodopsin-containing bacteria in the surface microlayer, water column, and epilithic biofilms of Lake Baikal was studied for the first time, employing *pufM* and rhodopsin genes, and compared to 16S rRNA diversity. We detected *pufM*-containing Alphaproteobacteria (orders Rhodobacterales, Rhizobiales, Rhodospirillales, and Sphingomonadales), Betaproteobacteria (order Burkholderiales), Gemmatimonadetes, and Planctomycetes. Rhodobacterales dominated all the studied biotopes. The diversity of rhodopsin-containing bacteria in neuston and plankton of Lake Baikal was comparable to other studied water bodies. Bacteroidetes along with Proteobacteria were the prevailing phyla, and Verrucomicrobia and Planctomycetes were also detected. The number of rhodopsin sequences unclassified to the phylum level was rather high: 29% in the water microbiomes and 22% in the epilithon. Diversity of rhodopsin-containing bacteria in epilithic biofilms was comparable with that in neuston and plankton at the phyla level. Unweighted pair group method with arithmetic mean (UPGMA) and non-metric multidimensional scaling (NMDS) analysis indicated a distinct discrepancy between epilithon and microbial communities of water (including neuston and plankton) in the 16S rRNA, *pufM* and rhodopsin genes.

## 1. Introduction

Photoheterotrophs are obligately heterotrophic bacteria capable of using light energy for ATP generation without fixing inorganic carbon and producing molecular oxygen. Photoheterotrophs include rhodopsin-containing bacteria [1] and aerobic anoxygenic phototrophs (AAPs) [1,2], using bacteriochlorophyll as a light-harvesting pigment. Photoheterotrophs lack RuBisCo, a key enzyme of a tricarbonic acid cycle, and therefore cannot fix CO_2_ for subsequent synthesis of organics [2,3,4,5]. AAPs and rhodopsin-containing bacteria are considered to play a crucial role in carbon cycling and energy flux in both marine and freshwater ecosystems [5,6,7,8].

These two types of photoheterotrophic bacteria have fundamentally different mechanisms of conversion of light energy to chemical energy. Photosynthetic apparatus of rhodopsin-containing bacteria is the simplest of all known, lacking an electron transport chain. It consists of a transmembrane protein, opsin, which is a light-driven proton pump, a chromophore (retinal), and ATP synthase [9].

Chlorophyll systems of AAPs are complex, being composed of multiple proteins, pigments, and cofactors forming reaction centers. The energy is passed along an electron transport chain [5,10]. AAPs likely evolved from the purple non-sulfur bacteria to fill an environmental niche, carrying out anoxygenic photosynthesis aerobically [4].

The first aerobic species of anoxygenic phototrophs were discovered in the 1970s (Aburatsubo Inlet, Pacific Ocean) [11]. The first freshwater AAP species, *Erythrobacter* (now *Sandaracinobacter*) *sibiricus* and *Erythromicrobium* (now *Erythromonas*) *ursincola*, were isolated from the surface of alkaline cyanobacterial mats growing in an alkaline thermal spring nearby Lake Baikal [12,13].

To date, the AAPs detected in different freshwater lakes and rivers belong to the class Alphaproteobacteria, orders Rhodospirillales, Rhodobacterales, Sphingomonadales, Rhizobiales, and Caulobacterales [10,14,15,16,17,18,19,20], as well as the class Betaproteobacteria, including genera *Limnohabitans* [16,18,19,21] and *Polynucleobacter* [22]. Additionally, the bacteria of phyla Gemmatimonadetes [23] and Firmicutes (Lactobacillales) [18] were reported. In contrast to marine ecosystems, Gammaproteobacteria are rare in freshwater bodies [10,22].

AAP bacterial cells are on average two-fold larger than the average bacterial cell size [24]. The use of light energy allows them to store more carbon (which would otherwise be respired) in the form of biomass [5]. Thus, AAPs likely have an ecological advantage over heterotrophs under conditions when bacterial growth is energy or carbon limited [25]. On the other hand, the larger size possibly makes AAP bacteria more subjected to protist grazing. AAPs can break down highly complex organics due to the activity of a broad range of proteins and enzymes. This makes them indispensable in carbon cycling in both saline and freshwater systems, as carbon available naturally is generally very complex both structurally and chemically for digestion by other microbes [24]. The AAPs in freshwater bodies comprised on average 1–7% or 37% maximum of total bacterial abundance [10,24], whereas in marine ecosystems, their share was 1–10%, up to 18% [10,26].

The recognition of microbial rhodopsins as light-driven ion pumps began in the 1970s when the photopigment named bacteriorhodopsin was found in *Halobacterium salinarum* (Archaea) [27]. Metagenomic studies revealed the presence of a new family of rhodopsins in marine bacteria, which was named proteorhodopsin [6]. Rhodopsin genes have been found in seawater, freshwater, and brackish water [22,28,29,30,31,32], and they are one of the most highly expressed and widely distributed proteins in marine bacterial communities [8]. The amount of rhodopsin-containing bacteria was 13–70% in marine ecosystems [9,31,33,34], and these were the dominant fraction among photoheterotrophs in freshwater lakes Damariscotta, Mendota, and Sparkling (USA) [22].

The diversity of rhodopsin-containing bacteria in freshwater ecosystems is poorly studied. The known dominant phylum is Actinobacteria [22,35,36]. Rhodopsins are also found in freshwater Flavobacteria, Beta-, Gamma- and Deltaproteobacteria, Verrucomicrobia, Sphingobacteria, and Alphaproteobacteria, including the SAR11 freshwater cluster [8,22,30].

The diversity of AAPs and rhodopsin containing bacteria in Lake Baikal has never been previously studied; this is the first work dedicated to this theme. Lake Baikal is of unique natural significance, being the oldest lake in the world with an age of ca. 25 million years. Taking into account that photoheterotrophs are a very active part of bacterial community playing an important role in carbon cycling and energy flux in freshwater ecosystems [5,6,7,8], it is essential to investigate those microorganisms for better understanding of microbial and biochemical processes occurring in Lake Baikal. The aim of the present work was to examine the diversity of aerobic anoxygenic phototrophs and rhodopsin-containing bacteria in different biotopes of Lake Baikal. We expected that photoheterotrophs community composition in Baikal water should be similar to other freshwater bodies, which was confirmed. We also hypothesized that photoheterotrophs community composition could differ in water and biofilms due to distinct living conditions, but those discrepancies eventually appeared to be mostly on the phylotype level.

## 2. Materials and Methods

Baikal is a dimictic oligotrophic lake with a surface area of 31,500 km^2^ and a water volume of 23,000 km^3^. Lake Baikal is conventionally divided into three basins: southern, central, and northern with maximum depths of about 1400, 1600, and 800 m, respectively. According to the content of major ions, water of the lake belongs to the bicarbonate class of the calcium group. The total dissolved solids content of Lake Baikal water is about 150 mg/L, which is very low [37].

**Figure 1:**
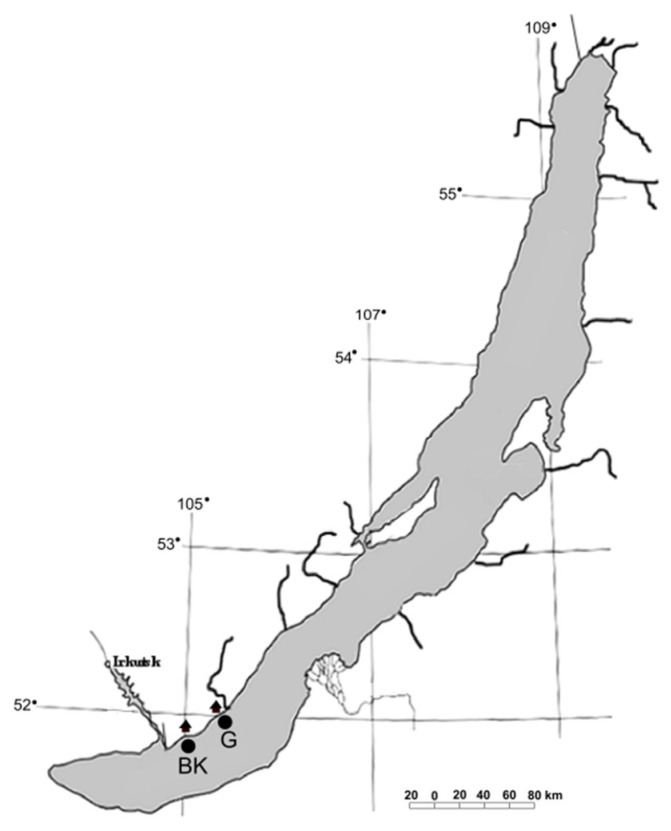
Sampling stations. BK—sampling station off the Bol’shiye Koty settlement; G—sampling station off the Bol’shoye Goloustnoye settlement.

The samples of surface microlayer (BK1, G1), water column (BK2, G2), bottom water (BK4, G4), and epilithic biofilms (BK51, BK53, G51, G52) were taken in the littoral zone of the southern basin of Lake Baikal off the Bol’shiye Koty settlement (51°53′93.0″ N; 105°03′83.8″ E) (BK) and off the Bol’shoye Goloustnoye settlement (52°01′36.5″ N; 105°24′11.8″ E) (G) in August 2019 (Figure 1). Surface microlayer was taken from a boat as described previously [38]. A metal mesh screen (26.5 cm in diameter) was horizontally submerged into the water and then horizontally lifted. After several seconds, the screen was bent and tilted to drain the water from the cells of the screen into a sterile container. Water column samples were taken from a depth of 5 and 10 m with a bathometer, then integrated. Stones with epilithic biofilms (two stones from each site) and samples of bottom water were taken by scuba divers off the Bol’shiye Koty settlement from a depth of 17 m and off the Bol’shoye Goloustnoye settlement from a depth of 12.3 m. The stones were placed into sterile plastic containers fulfilled with surrounding water to prevent drying. Bottom water was collected into sterile plastic bottles.

1 cm^2^ of epilithic biofilms from each stone was scraped off with a sterile scalpel and freezed until further analysis. Samples of water (1 L) were filtered through polycarbonate membrane filters (pore size 0.22 μm) (Millipore, Burlington, MA, USA). Total DNA was extracted using the phenol-chlorophorm method [39]. Extracted DNA samples were equimolarly mixed and used as a template for the analysis.

PO_4_^3−^, NO_3_^−^, NO_2_^−^, and NH_4_^+^ were analyzed in water samples filtered through mixed cellulose ester membrane filters (Advantec, Tokyo, Japan, pore diameter 0.45 µm). The content of total organic carbon (TOC) was determined in unfiltered water. Concentration of dissolved nutrients was determined using a photoelectric colorimeter (KFK-3-01-ZOM3, Zagorskii optiko-mekhanicheskii zavod [Zagorskii optical mechanics factory], Sergiev Posad, Russia) according to Wetzel and Likens [40]. PO_4_^3−^ was identified by the Denigès–Atkins method in modification with tin chloride. NH_4_^+^ was detected by the indophenol method [41]. NO_3_^−^ and NO_2_^−^ content was measured by high performance liquid chromatography (EcoNova, Novosibirsk, Russia) with UV detection on an inverse-phase column modified with octadecyltrimethylammonium bromide [42]. Total organic carbon was determined by a total carbon/nitrogen analyzer (Vario TOC cube, Elementar, Langenselbold, Germany).

The samples of epilithic biofilms were scraped off with a sterile scalpel from each stone and dried before further analysis. Biofilms taken in the same sampling site were integrated (BK51 + BK53 and G51 + G52, respectively). TOC and N total were determined by element analyzer (Flash EA 1112 CHNS, Thermo Fisher Scientific, Waltham, MA, USA) in Baikal Analytical Collective Instrumental Center (A.E. Favorsky Irkutsk Institute of Chemistry SB RAS). P total was determined by the persulfate oxidation method [43,44,45].

Physical and chemical characteristics of water samples and biofilms are provided in Table 1 and Table 2.

Fragments of the 16S rRNA gene containing two variable regions V3–V4 were amplified using eubacterial primers 343F (forward) (CTCCTACGGRRSGCAG) and 806RB (reverse) (GGACTACNVGGGTWTCTAAT) [46,47]. BioMaster HS-Taq PCR-Color (2x) kit (Biolabmix, Russia) was used to prepare the reaction mix. PCR conditions were as follows: an initial denaturation step at 94 °C for 9 min 50 s and 25 cycles at 94 °C (30 s), 58 °C (30 s), 72 °C (45 s), and a final elongation step at 72 °C for 8 min. The majority of environmental studies employ the *pufM* gene (encoding the M subunit of the bacterial reaction center) as a marker for anoxygenic phototrophs harboring type-2 reaction centers [1,48,49]. Fragments of the *pufM* gene (~220 bp fragments) were amplified using primers *pufM*_uniFfresh (forward) (GGNAAYYTGTTYTAYAACC) and *pufM*_uniRfresh (reverse) (CCCATSGTCCANCKCCARAA) [22,50]. PCR conditions were as follows: an initial denaturation step at 95 °C for 5 min and 40 cycles at 94 °C (20 s), 50 °C (60 s), 72 °C (60 s), and a final elongation step at 72 °C for 10 min. Fragments of the rhodopsin gene (KEGG ID: "K04250") (~345 bp fragments) were amplified using primers PRpf2 (forward) (TAYCGYTAYGTNGAYTGG) and PRpr2 (reverse) (ATYGGRTANACRCCCCA) [22,36]. PCR conditions were as follows: an initial denaturation step at 95 °C for 5 min and 40 cycles at 94 °C (20 s), 46 °C (60 s), 72 °C (45 s), and a final elongation step at 72 °C for 10 min. Amplicon libraries were generated with gene-specific primers, prepared for sequencing with Nextera XT kit (Illumina, San Diego, CA, USA) and sequenced using MiSeq (Illumina, USA) (SB RAS Genomics Core Facility, Novosibirsk, Russia). Results were uploaded into NCBI SRA (PRJNA613961).

Assessment of amplicon libraries quality was performed with FastQC software (http://www.bioinformatics.babraham.ac.uk/projects/fastqc Last access date: 14 April 2021), primers and spurious sequences were trimmed using cutadapt v1.14 [51]. Metagenomic data for the G52 sample (16S rRNA gene amplicon library) were of poor quality and were not taken into further analysis. Metagenomic data were processed using DADA2 software (parameters: maxN = 0, maxEE = c(2,4)), including alignment, filtering for chimeras, short and spurious sequences, and exact sequence variants (ESVs) clustering [52]. 16S rRNA gene fragments were aligned and taxonomically assigned using SILVA v.132 database with a confidence threshold of 80% [53] and clustered to operational taxonomic units (OTUs) at 0.03 distance with mothur v.1.40.0 [54].

Nucleotide sequences of functional genes fragments were translated into amino acid sequences, then aligned and taxonomically assigned using BLASTp algorithm against NCBI-nr database. After that, sequences were clustered at 100% of amino acid sequence similarity for further analysis.

Maximum Likelihood tree for 16S rRNA gene fragments alignment was built using MEGA X software tool [55] with bootstrap sampling and using K2P + G + I substitution model based on jmodeltest tool results [56]. Phylogenetic trees for functional gene alignments were computed following the Bayesian Markov chain Monte Carlo (MCMC) method using BEAST v1.10.4 [57] with HKY + G + I substitution model. MCMC was run for 2 million steps. The output was analyzed in Tracer v1.7.1 [58] and burn-in was adjusted to attain an appropriate effective sample size (ESS) more than 200.

Statistical analysis was performed with the vegan package [59] using the R language [60]. The rarefaction curves were plotted to evaluate the sufficiency of the sequencing depth (Supplementary Materials Figure S1). We used sub-sampled by smallest value data set for further analysis of alpha- and beta-diversity. Alpha-diversity was analyzed using the Chao1, Shannon, and Simpson indices. Beta-diversity was analyzed using unweighted pair group method with arithmetic mean (UPGMA) and non-metric multidimensional scaling (NMDS) methods based on Bray-Curtis dissimilarity metrics. Analysis of variance (*PERMANOVA*/“*adonis*” function from *vegan* package) was used to compare samples by sampling site and biotope (Table S1).

## 3. Results

### 3.1. 16S rRNA

**Figure 2:**
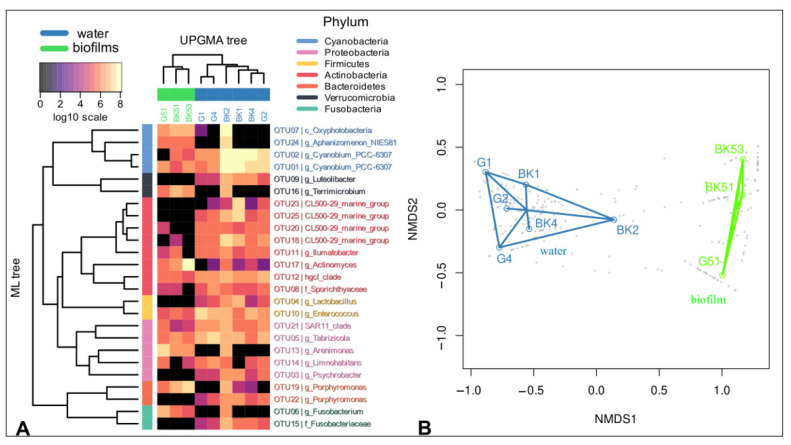
Similarities and differences between epilithon (G51, BK51, BK53), neuston (BK1, G1), and plankton (BK2, BK4, G2, G4) of Lake Baikal. Heatmap of 25 most represented OTUs_0.03_ of 16S rRNA gene, UPGMA dendrogram and phylogenetic tree (**A**) and NMDS plot (**B**) based on the Bray-Curtis similarity matrix. Blue lines highlight water cluster of samples; green lines highlight epilithic biofilms cluster of samples. BK—samples taken off the Bol’shiye Koty settlement; G—samples taken off the Bol’shoye Goloustnoye settlement.

After sequencing and primary analysis, 169,497 sequences with an average read length of 418 bp were kept for downstream analysis. The number of reads in the samples ranged from 8893 to 49,218. Overall, we detected 1217 ESVs grouped in 810 OTUs with a cluster distance of 0.03. Rarefaction curves constructed from OTU_0.03_ (Figure S1) showed sufficient sequencing depth for all the samples. UPGMA and NMDS analysis indicated that the sampling site, as well as a biotope, significantly influences the species composition of microbiomes (Figure 2A,B). Microbial communities of epilithic biofilms and water (including surface microlayer, water column, and bottom water) formed distinct clusters. Microbial communities of the surface microlayer, water column, and bottom water did not have significant differences (PERMANOVA).

**Figure 3:**
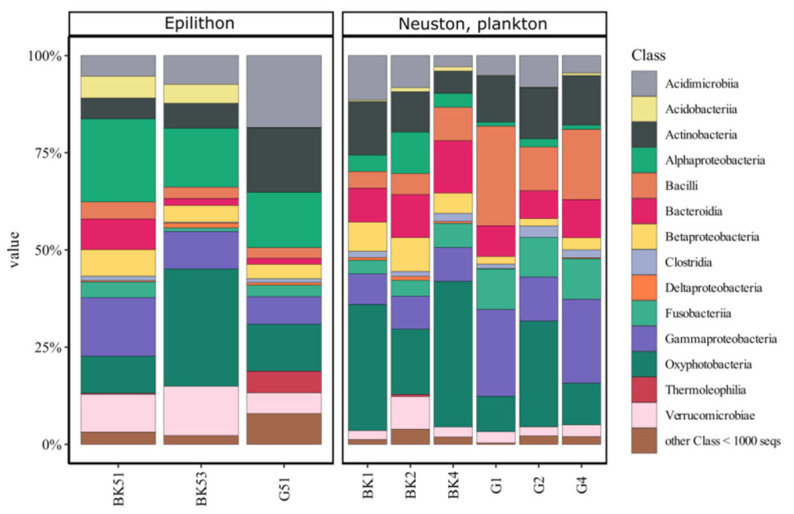
Taxonomic identification of 16S rRNA bacterial reads of epilithon (G51, BK51, BK53), neuston (BK1, G1), and plankton (BK2, BK4, G2, G4) of Lake Baikal at the class level.

Cyanobacteria, Actinobacteria, Proteobacteria, Bacteroidetes, Verrucomicrobia, Firmicutes, and Fusobacteria dominated the studied microbiomes. The representatives of these phyla comprised more than 1% of the total number of sequences in each microbial community (Table S2). The minor phyla included Acidobacteria, Armatimonadetes, Chloroflexi, Deinococcus-Thermus, Dependentiae, Gemmatimonadetes, Nitrospirae, Patescibacteria, and Planctomycetes. At the same time, bacterial communities of the surface microlayer (neuston), water column, and bottom water (plankton) significantly differed from the epilithic biofilms microbiomes (epilithon) in the representation of dominant taxa. Alphaproteobacteria, Verrucomicrobia, and Acidobacteria were much more abundant in epilithon than in neuston and plankton. Cyanobacteria, Firmicutes, Bacteroidota, and Fusobacteria prevailed in neuston and plankton. Actinobacteria were almost equal in the microbiomes of water and epilithic biofilms (Figure 3, Table S2).

The taxonomic composition of epilithon and water microbiomes also differed at the genus and family level. Among Alphaproteobacteria, Rhizobiales and Rhodobacterales (*Tabrizicola*) were abundant in epilithon compared to water microbiomes, whereas the SAR11 cluster (Pelagibacterales) was exclusively represented in neuston and plankton. *Rhodoferax*, *Leptothrix,* and *Methylotenera* (Betaproteobacteria) were more abundant in epilithon, *Limnohabitans* was detected exceptionally in neuston and plankton. Composition and structure of cyanobacterial communities in Lake Baikal neuston (BK1, G1), plankton (BK2, BK4, G2, G4), and epilithon (G51, BK51, BK52) were described by Belykh et al. (2020) [61]. The *Synechococcus*/*Cyanobium* cluster and *Dolichospermum lemmermannii* represented neustonic and planktonic Cyanobacteria, whereas *Tychonema* sp., *Phormidium* sp., *Symplocastrum* sp., *Pseudanabaena* sp., and *Synechococcus* sp. benthic Cyanobacteria. Actinobacteria also included “typical water phylotypes”: hgcI_clade (Sporichthyaceae) and CL500-29_marine_group (Ilumatobacteraceae).

### 3.2. Aerobic Anoxygenic Phototrophs

After sequencing and primary analysis, we obtained 485,240 sequences of *pufM* gene fragment with an average read length of 191 bp. In total, we detected 1128 ESVs representing 173 unique amino acid sequences. Based on BLASTp, similarity with the closest homologues ranged from 85 to 100%.

**Figure 4:**
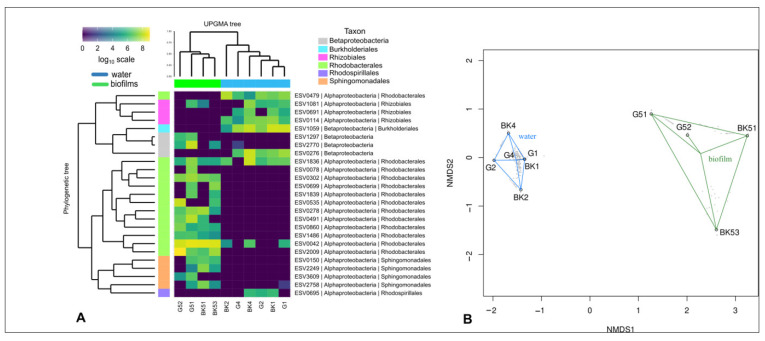
Similarities and differences between AAPs of epilithon (G51, G52, BK51, BK53), neuston (BK1, G1), and plankton (BK2, BK4, G2, G4) of Lake Baikal. Heatmap of 25 most represented ESVs of *pufM* gene, UPGMA dendrogram and phylogenetic tree (**A**) and NMDS plot (**B**) based on the Bray-Curtis similarity matrix. Blue lines highlight water cluster of samples; green lines highlight epilithic biofilms cluster of samples. BK—samples taken off the Bol’shiye Koty settlement; G—samples taken off the Bol’shoye Goloustnoye settlement.

UPGMA and NMDS analysis revealed that biotope significantly influenced AAP community composition (Figure 4A,B). AAPs detected in the epilithic biofilms and the water (including surface microlayer, water column, and bottom water) formed distinct clusters. AAP communities of the surface microlayer, water column, and bottom water did not have significant differences (PERMANOVA).

**Figure 5:**
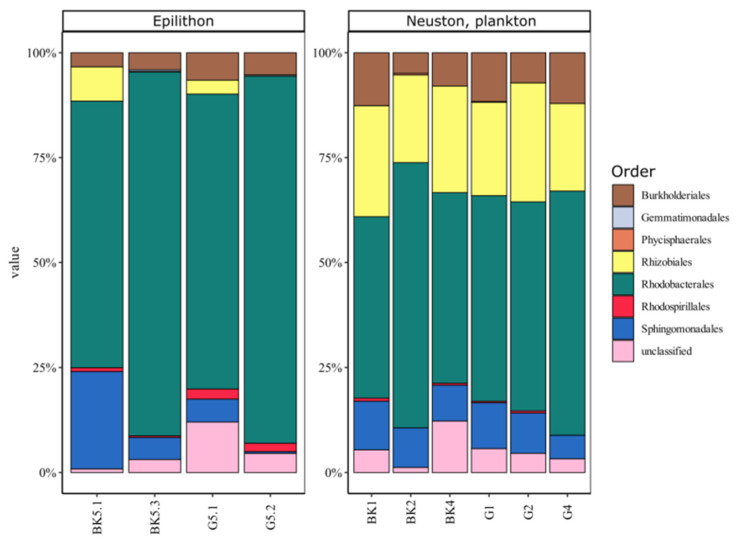
Taxonomic identification of *pufM* gene reads of epilithon (G5.1, G5.2, BK5.1, BK5.3), neuston (BK1, G1), and plankton (BK2, BK4, G2, G4) of Lake Baikal at the order level. BK—samples taken off the Bol’shiye Koty settlement; G—samples taken off the Bol’shoye Goloustnoye settlement.

Sequences of the *pufM* gene fragments were mainly identified to the order level due to the relatively short *pufM* amplicon and paucity of reference sequences. We detected Alphaproteobacteria (Rhodobacterales, Rhizobiales, Rhodospirillales, and Sphingomonadales), Betaproteobacteria (Burkholderiales), Gammaproteobacteria, as well as phyla Gemmatimonadetes and Planctomycetes (class Phycisphaerae). Rhodobacterales dominated both biofilm and water communities of Lake Baikal, whereas Rhizobiales and Burkholderiales were more represented in neuston and plankton compared to epilithon (Figure 5). Gemmatimonadetes were detected only in the water, but Phycisphaerae and Gammaproteobacteria exclusively in the epilithic biofilms. Some phylotypes were identified to the genus level with a similarity of 98 to 100% (Table 3). According to Chao1, Shannon, and Simpson indices, alpha diversity was comparable in all the studied biotopes (Table 4).

### 3.3. Rhodopsin-Containing Bacteria

After sequencing and primary analysis, we obtained 57716 sequences of a rhodopsin gene fragment with an average read length of 327 bp. In total, we detected 99 ESVs consisting of unique amino acid sequences. Based on BLASTp, similarity with the closest homologue ranged from 66 to 99%.

**Figure 6:**
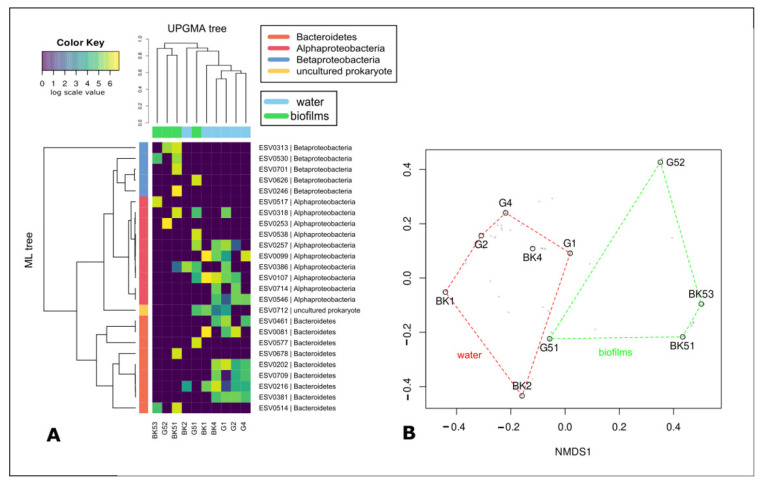
Similarities and differences between epilithon (G51, G52, BK51, BK53), neuston (BK1, G1), and plankton (BK2, BK4, G2, G4) of Lake Baikal. Heatmap of the 25 most represented ESVs of rhodopsin gene, UPGMA dendrogram and phylogenetic tree (**A**) and NMDS plot (**B**) based on the Bray-Curtis similarity matrix. Red lines highlight water cluster of samples; green lines highlight epilithic biofilms cluster of samples. BK—samples taken off the Bol’shiye Koty settlement; G—samples taken off the Bol’shoye Goloustnoye settlement.

UPGMA and NMDS analysis indicated that biotope significantly biased rhodopsin-containing bacteria community composition at the phylotype level (Figure 6A,B). Rhodopsin-containing bacteria detected in the epilithic biofilms and the water formed distinct clusters, except for the G51 biofilm microbiome, which clustered with plankton communities. Rhodopsin-containing bacterial communities of the surface microlayer, water column, and bottom water did not have significant differences (PERMANOVA).

**Figure 7:**
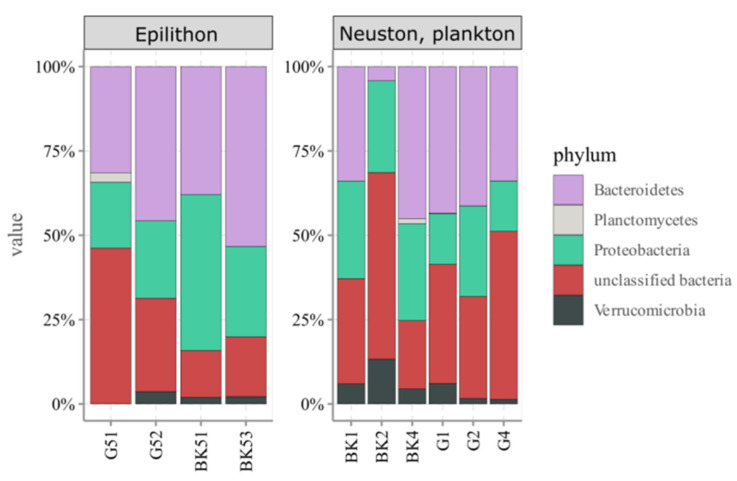
Taxonomic identification of rhodopsin gene reads of epilithon (G51, G52, BK51, BK53), neuston (BK1, G1), and plankton (BK2, BK4, G2, G4) of Lake Baikal at the phylum level. BK—samples taken off the Bol’shiye Koty settlement; G—samples taken off the Bol’shoye Goloustnoye settlement.

Taxonomic identification was performed only to the phylum level due to the paucity of reference sequences. Rhodopsin-containing bacteria of Lake Baikal belonged mainly to the phyla Bacteroidetes and Proteobacteria. Planctomycetes and Verrucomicrobia were a minor fraction (Figure 7). All these phyla were evenly represented in the water microbiomes and epilithon. The number of sequences unidentified to the phylum level was high: 29% in the water microbiomes and 22% in the epilithon. A few phylotypes were identified to the genus level with a similarity of 97 to 99% (Table 5). According to Chao1, Shannon, and Simpson indices, alpha diversity was comparable in all the studied biotopes (Table 6).

## 4. Discussion

The main goal of our research was to assess the diversity of AAPs and rhodopsin-containing bacteria in Lake Baikal as it has never been done before. The method of sequencing of functional genes amplicons which was applied allowed us to get the first insight into the diversity of Baikal photoheterotrophs and to compare it with other freshwater bodies studied. Interesting data was obtained regarding differences between epilithon and water photoheterotrophic bacterial communities; the diversity of AAPs and rhodopsin-containing bacteria inhabiting those biotopes has not been compared, to the best of our knowledge.

16S rRNA gene diversity of Lake Baikal bacterioneuston and bacterioplankton communities was similar to that described by Galach’yants et al. (2017), Mikhailov et al. (2015, 2019), and Kurilkina et al. (2016) [62,63,64,65]. The dominant phyla in Lake Baikal neuston and plankton were Cyanobacteria, Actinobacteria, Proteobacteria, Bacteroidetes, and Verrucomicrobia. In the current work, Firmicutes and Fusobacteria were also referred to as the major phyla. The other phyla detected were Planctomycetes, Acidobacteria, Armatimonadetes, Chloroflexi, Deinococcus-Thermus, Gemmatimonadetes, Nitrospirae, and Firmicutes [62,63,64,65]. These phyla are known to be common in all freshwater bodies [66]. 16S rRNA gene sequences of Lake Baikal bacteria had high homology with the sequences of bacteria inhabiting other freshwater bodies all over the world. This fact confirms the similarity of microbial communities of freshwater ecosystems [67].

Biofilms are major sites of carbon cycling and ecosystem productivity in freshwater ecosystems, even in the world’s largest lakes [68,69]. Taxonomic composition of Lake Baikal epilithic biofilms was observed by Parfenova et al. (2013) and Sorokovikova et al. (2013) [70,71]. Phyla diversity was comparable to that described in the current work. Cyanobacteria, Proteobacteria, and Bacteroidetes dominated; Actinobacteria, Verrucomicrobia, Planctomycetes, Acidobacteria, Chloroflexi, Gemmatimonadetes, Nitrospirae, and Firmicutes were present as well. Similar composition of bacterial phyla in epilithic biofilms of oligotrophic mountain lakes was shown by Bartrons et al. (2012) [72]. Recently, community structure of river biofilms was estimated by Romero et al. (2020) [73]. Phyla composition turned out to be much the same as previously described. The most represented phyla were Proteobacteria, Bacteroidetes, Cyanobacteria, and Firmicutes.

The proportion of Cyanobacteria in the total number of sequences was big and averaged 23% in neuston and plankton and 17% in the epilithon. Cyanobacteria are a big part of Lake Baikal autotrophic picoplankton (more than 90% of its abundance), playing a key role in freshwater oligotrophic ecosystems as a considerable source of primary production [74,75]. Cyanobacteria convert carbon dioxide and water into organic matter during photosynthesis and release oxygen, making the existence of heterotrophic organisms that consume organic substances and aerobic organisms that require oxygen possible. Picoplankton species of the cluster *Synechococcus*/ *Cyanobium* and *Dolichospermum lemmermannii* mainly represented cyanobacteria in neuston and plankton. Benthic and periphyton species, *Synechococcus* sp., *Calothrix* sp., *Tychonema* sp., and *Pseudanabaena* sp., were abundant in the biofilms. One of the dominant picoplankton species was *Dolichospermum lemmermannii* known to produce paralytic mollusc toxins (saxitoxins) harmful for human beings and mammals [76].

Actinobacteria comprised 19% of all sequences in neuston and plankton and 22% in epilithon. Actinobacteria are ubiquitous in the epilimnion of freshwater bodies [66]. These bacteria are chemo-organoheterotrophic microorganisms, at the same time possessing rhodopsin pigment allowing them to acquire the supplementary ATP from the solar light [66]. Actinobacteria are free living bacteria with a small size of cells, which helps them to escape grazing. All these features enable dominating of Actinobacteria in different water bodies. In neuston and plankton, OTUs 8, 12, 17, 18, 23, and 25 (Acidobacteriales, Frankiales, and Microtrichales not identified at the genus level) prevailed, whereas OTUs 11, 20, 32, and 33 (Microtrichales, Propionibacteriales, and Frankiales not identified at the genus level) dominated epilithon.

Proteobacteria was the most represented phylum in all the biotopes, which included 24% of neuston and plankton and 33% of epilithon sequences. Proteobacteria are ubiquitous microorganisms, but freshwater bodies are usually dominated by Betaproteobacteria [66]. These are copiotrophs growing fast in the excess of organics. In Lake Baikal, the most represented Betaproteobacteria genus was *Limnohabitans*; it was detected exclusively in neuston and plankon. The genus *Limnohabitans* (Burkholderiales, Betaproteobacteria) is a common and highly active component of freshwater bacterioplanktonic communities [66]. *Limnohabitans* is capable of consuming phytoplankton-derived DOC and, thus, plays an important role in the carbon cycle in freshwater bodies [77]. In the biofilms, dominant phylotypes were assigned as *Rhodoferax*, *Methylotenera,* as well as Burkholderiaceae not identified at the genus level. *Rhodoferax* are purple non-sulfur bacteria common in biofilms capable of both living with or without oxygen acting as photoautotrophs [78]. *Methylotenera* are representatives of methylotrophs, microbes capable of utilizing single C_1_ compounds as sole sources of energy and carbon [79]. These bacteria are also effective degraders of complex organic compounds [79].

Among Alphaproteobacteria, the most represented OTU was assigned as the SAR11 cluster (Pelagibacterales). It was detected only in neuston and plankton. These bacteria are widely distributed in oligotrophic water bodies and are one of the most abundant microorganisms on the Earth [66,80]. In the epilithon, OTU 6 (*Tabrizicola*) and OTU 63 (*Polymorphobacter*) were the most represented phylotypes. Some species of the genera *Tabrizicola* and *Polymorphobacter* produce bacteriochlorophyll *a* under aerobic heterotrophic conditions and possess *pufLM* photosynthesis-related genes [20,81].

Bacteroidetes comprise a considerable part of bacterial community in the epilimnion of lakes [66]. In our work, 10% of neuston and plankton sequences and 4% of epilithon sequences belonged to that phylum. These bacteria can attach to the particles and play an important role in the degradation of complex biopolymers. In water bacterial communities, the most represented genera were *Flavobacterium* and *Algoriphagus*. In the epilithon, *Flavobacterium* dominated as well. Bacteria of the genus *Flavobacterium* are one of the most numerous Bacteroidetes representatives in freshwater bodies acting as copiotrophs [66]. Members of the genus *Algoriphagus* are saccharolytic bacteria initially isolated from algal-rich biotopes [82].

Verrucomicrobia are also presented in all freshwater lakes. These bacteria have been observed in both surface and hypolimnetic waters, suggesting a variety of metabolic strategies within the group [83,84]. In Lake Baikal, they were presented mainly in the biofilms (9% of sequences). In neuston and plankton, sequences belonging to that phylum comprised 5%. In water bacterial communities, OTU 9 (*Luteolibacter*) was the most represented; the same OTU prevailed in the epilithon, but its proportion was higher compared to neuston and plankton. *Luteolibacter* strains are chemo-organoheterotrophic bacteria showing a nutritional preference for simple sugars and complex protein substrates [85].

In our study, Firmicutes was the major phylum as well. It was mostly represented by allochthonous microorganisms belonging to the genera *Lactobacillus* and *Enterococcus*, typical representatives of gut microbiome [86], showing fecal contamination of water. This was probably due to the localization of sampling stations off the settlements not equipped by sewage treatment plants. Ships significantly contribute to the fecal contamination as well [87].

Representatives of the phylum Fusobacteria were abundant in our samples as well. These are also members of gut microbiome [86] and confirm fecal contamination of the water.

Thus, the members of Lake Baikal water and epilithic biofilms bacterial communities are active participants of carbon cycle and energy flux in the lake being essential to the maintenance of ecosystem functioning. Cyanobacteria perform primary production converting inorganic carbon into organic compounds using solar energy along with phytoplankton. Other members of the community are active degraders of complex organic matter being aerobic chemo-organoheterotrophs. Some bacteria are well adapted to the oligotrophic conditions due to possessing additional mechanism of energy harvesting, such as photoheterotrophs.

The taxonomic composition of AAP communities in Lake Baikal is similar to that in other freshwater bodies [14,15,16,18,22,88,89]. As in Lake Baikal, Rhodobacterales and Burkholderiales dominated, and Rhizobiales, Rhodospirillales, and Sphingomonadales were detected almost in every studied water body.

*pufM*-containing Planctomycetes (class Phycisphaerae) were also detected in Lake Baikal. Planctomycetes are environmentally important bacteria that are key players in global carbon and nitrogen cycles. Planctomycetes seem to be associated primarily with particles, surfaces, microbial mats, and biofilms while they can be very abundant in other habitats as well [90]. The closest *pufM* homologue of Baikal representatives (MBC7770036, 98% similarity) was detected in a high arctic glacier in Northeast Greenland [91]. *pufM*-containing Planctomycetes were also reported in the South China Sea [92].

AAPs identified to the genus level belonged to the genera *Tabrizicola*, *Erythrobacter*, *Blastomonas*, and *Sphingomonas*.

The closest *pufM* homologues of the Baikal representatives of *Erythrobacter*, *Blastomonas* and *Sphingomonas* were detected in freshwater bodies. *Erythrobacter* sp. HU12-14 (AGK27892, 98–100% similarity) was isolated from Lake Hulun (Inner Mongolia); *Sphingomonas* sp. H151 (AGK27878, 100% similarity) and *Blastomonas* sp. Y27 (AGK27877, 98–100% similarity) from Lake Yongxinghu (Inner Mongolia). These genera are known to include aerobic anoxygenic phototrophs [93,94,95]. In Lake Baikal, phototrophic *Erythrobacter*, *Blastomonas*, and *Sphingomonas* were detected mainly in the epilithon.

Type species of the genus *Tabrizicola* were isolated from different freshwater and saline ecosystems [96,97,98]. Since this genus has been discovered not long ago, its ecology is poorly studied. Notably, the phototrophic *Tabrizicola* were rather abundant in Lake Baikal (4% of all *pufM* gene sequences) and were detected exclusively in epilithon. The closest homologue, *Tabrizicola* sp. (QBQ34549, 98–100% similarity), was detected in a lake on Tibetan Plateau.

**Figure 8:**
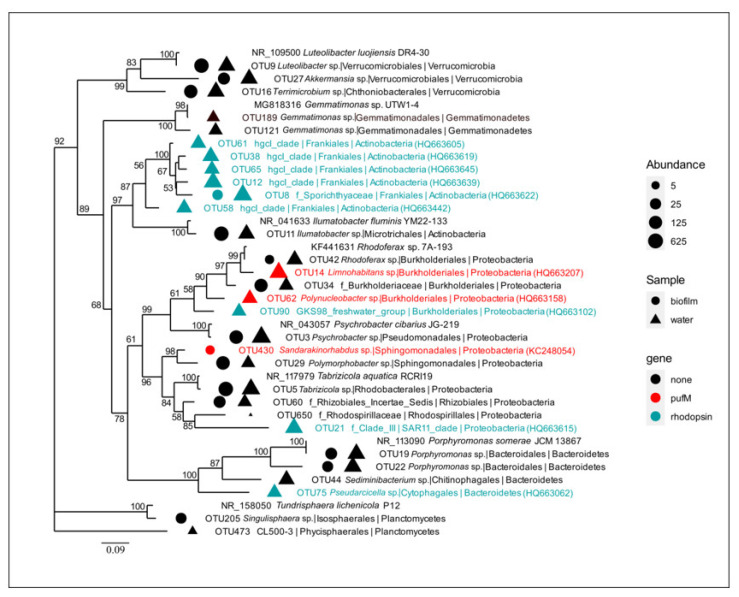
Maximum-Likelihood tree of 16S rRNA gene alignment with the closest homologues for the most abundant and potentially phototrophic OTUs. Circles and triangles are representing biotope type and colors are showing presence (blue and red) or absence (black) of the functional gene in reference organism (similarity 99–100%). The size of the circle/triangle corresponds to the number of sequences belonging to the OTU in the epilithic biofilm/water microbial communities, respectively.

Given that *pufM* gene sequences were identified predominantly only to the order level, we reviewed the 16S rRNA diversity of hypothetically AAP bacteria in neuston, plankton, and epilithon (Figure 8). The AAP genera, *Roseomonas* (Acetobacterales), *Bradyrhizobium* (Rhizobiales), *Tabrizicola* (Rhodobacterales), *Blastomonas* (Sphingomonadales), *Porphyrobacter* (Sphingomonadales), *Sandarakinorhabdus* (Sphingomonadales), *Sphingomonas* (Sphingomonadales), *Polymorphobacter* (Sphingomonadales), *Polynucleobacter* (Burkholderiales), and *Limnohabitans* (Burkholderiales), were detected in the studied biotopes of Lake Baikal (totally 10138 16S rRNA gene sequences, 6% of all obtained sequences). *Limnohabitans* and *Tabrizicola* dominated. Moreover, we detected the *Rhodobacter* and *Rhodoferax* representatives. These are purple non-sulfur bacteria capable of photolithoautotrophy in anaerobic conditions with the availability of light [78]. In aerobic conditions, they function as chemoorganoheterotrophs.

Recently, the photosynthesis genes *pufLM* and *bchY* from the *Limnohabitans* representatives were detected [21]. Now it is known that *Limnohabitans* comprises a big part of the AAP community in freshwater ecosystems [21,89]. A considerable amount (1.8%) of our 16S rRNA sequences was identified as *Limnohabitans*. All of them were detected in the water biotopes: surface microlayer, water column, and bottom water. There were no *Limnohabitans* bacteria in the epilithon. The closest homologue, betaproteobacterium SCGC AAA027-O07 (HQ663710, 99.53% similarity), possessed the *pufM* gene; therefore, the Baikal representatives, probably, also had this gene (Figure 8).

UPGMA and NMDS analysis indicated that biotope significantly biased the taxonomic composition of AAP bacteria. Water microbiomes and epilithon had distinct differences at the phylotype level (Figure 4A,B). There were “typical epilithon phylotypes” and “typical water phylotypes”. AAPs belonging to Rhodobacterales dominated all biotopes. Rhizobiales and Burkholderiales were abundant in neuston and plankton in contrast to epilithon (Figure 5). Phototrophic *Tabrizicola* and *Erithrobacter* were detected only in the epilithic biofilms, representing “typical epilithon phylotypes”.

The information about AAPs in epilithic biofilms is scarce; we managed to find only one paper dedicated to this theme. Hirose et al. (2016) investigated aerobic anoxygenic phototrophs in epilithic biofilms of the Tama River (Japan) [17]. Rhodospirillales (*Roseomonas*), Rhodobacterales (*Tabrizicola*) and Sphingomonadales (*Polymorphobacter*, *Sandarakinorhabdus*, *Sphingomonas*) were detected there [17] as in the Baikal epilithic biofilms. Remarkably, the closest homologue of one Baikal *Tabrizicola* phylotype, *Rhodobacteraceae* bacterium W19 (LC094483, 100% similarity), was detected in epilithic biofilms of the Tama River [17]. The AAP community of epilithic biofilms in Lake Baikal also included Burkholderiales and Gemmatimonadetes in contrast to the Tama River.

Rhodopsin-containing bacteria in freshwater bodies are poorly studied. The diversity of phyla of this phototrophic group in Lake Baikal is comparable to other investigated freshwater bodies. Based on BLASTp analysis, the rhodopsin-containing bacterial community of Lake Baikal included phylum Planctomycetes (Figure 7). The closest homologue was detected in the sea water, off the coast of Alicante (Spain) (RZO64088, 72% similarity) [99]. Rhodopsin-containing Planctomycetes were also detected in littoral microbial mats of high latitude freshwater lakes (Canada) [100].

Thus, there were detected both *pufM*-containing and rhodopsin-containing Planctomycetes in Lake Baikal. According to Zeng et al. (2020), “there is emerging genomic evidence that (bacterio-)chlorophyll- and proton-pumping rhodopsin-based phototrophic systems can coexist in a single bacterium” [91]. At the same time, due to the fact that *pufM*- and rhodopsin-containing Planctomycetes in Lake Baikal had different closest homologues, we can propose that the type of phototrophic system might depend on the distinct class, order, family, genus, or species affiliation of Planctomycetes strain, just like in Proteobacteria. In Proteobacteria, there are representatives of aerobic anoxygenic phototrophs [16,17,18,19,20,21,22] as well as rhodopsin-containing bacteria [29,30,100].

Actinobacteria are known to be the most abundant rhodopsin-containing bacteria in previously studied freshwater ecosystems [22,35,36]. In Lake Baikal, they were not detected. Nevertheless, 16S rRNA diversity analysis revealed that Actinobacteria comprised 21% of all obtained sequences and were equal in epilithon, neuston, and plankton. Among them, 26% had close homologues (100% similarity) possessing the rhodopsin gene, especially, Actinobacterium SCGC AAA280-O03 (HQ663639, 100% similarity with OTU 10) (Figure 8). Therefore, the Baikal representatives could also have this gene. Most likely, the Actinobacteria rhodopsin gene sequences were among those unidentified to the phylum level. According to the 16S rRNA data, Actinobacteria were a considerable part of the rhodopsin-containing phototrophic community of Lake Baikal.

Freshwater representatives of the SAR 11 cluster (Pelagibacterales) are also known to have rhodopsin [29,30]. 16S rRNA sequences of the SAR 11 bacteria were detected in neuston and plankton of Lake Baikal (1.1% of all sequences) but not in epilithon. The closest homologue was the rhodopsin-containing strain Alphaproteobacterium SCGC AAA 280-B11 (HQ663835, 100% similarity) [22] (Figure 8). We suppose that the Baikal SAR 11 bacteria also might possess a rhodopsin gene.

Data regarding the diversity of rhodopsin-containing bacteria in epilithic biofilms are scarce. Vigneron et al. (2018) studied genes coding for microbial rhodopsins in littoral microbial mats of high latitude freshwater lakes (Canada) [100]. Rhodopsin genes were affiliated to Actinobacteria, Acidobacteria, Bacteroidetes, Cyanobacteria, Planctomycetes, and Alpha- and Betaproteobacteria. Epilithon of Lake Baikal included the same phyla detected in neuston and plankton: Bacteroidetes, Proteobacteria, Verrucomicrobia, and Planctomycetes. Nevertheless, UPGMA and NMDS analysis indicated significant differences between water microbiomes and epilithon at the phylotype level (Figure 6A,B). Similar to the AAP bacterial communities, there were “typical epilithon phylotypes” and “typical water phylotypes”. For example, OTUs 1901, 2299 (Bacteroidetes, Cytophagales, *Aquirufa*) and 1360 (Bacteroidetes, Cytophagales, *Runella*) were exclusively in neuston and plankton, whereas OTU 678 (Bacteroidetes, Cytophagales, *Runella*) only in epilithon (Table 5).

The results showed that epilithon differed from neuston and plankton mainly at the genus and phylotype level; taxonomic composition of microbial communities on the higher levels was pretty similar. That was true for 16S rRNA gene as well as for *pufM* and rhodopsin. It was an interesting finding needed to be explained. It is well known that there are free-living bacteria and attached forms, which need a substrate to adherence to. These two types of bacteria differ taxonomically at the phylotype level [101]. The first type is obviously detected in plankton, and the second on the surfaces including stones. The second type takes part in the forming of epilithic biofilms. That is why epilithon and plankton in Lake Baikal were taxonomically distinct at the genus and phylotype level.

## 5. Conclusions

Thus, we studied the diversity of *pufM*- and rhodopsin-containing bacteria in neuston, plankton and epilithon of Lake Baikal for the first time. AAPs and rhodopsin-containing phototrophs were ubiquitous, detected in all studied biotopes located in euphotic zone. These bacteria are well adapted to oligotrophic conditions of Lake Baikal possessing an additional mechanism of energy harvesting.

Like in previously studied freshwater bodies, the *pufM*-containing bacterial community in Lake Baikal included Alphaproteobacteria (Rhodobacterales, Rhizobiales, Rhodospirillales, Sphingomonadales), Betaproteobacteria (Burkholderiales), Gammaproteobacteria, Gemmatimonadetes, and Planctomycetes. Rhodobacterales dominated all studied biotopes in Lake Baikal.

The diversity of phyla of rhodopsin-containing bacteria is comparable to that in other studied freshwater ecosystems. Bacteroidetes and Proteobacteria prevailed in all studied biotopes of Lake Baikal, Verrucomicrobia and Planctomycetes were detected as well. According to the 16S rRNA data, Actinobacteria were also a considerable part of the rhodopsin-containing phototrophic community in Lake Baikal. A lot of rhodopsin gene sequences detected in epilithon (22%) as well as in neuston and plankton (29%) were not identified even to the phylum level. The diversity of rhodopsin-containing bacteria in the epilithic biofilms was comparable to that in neuston and plankton at the phylum level.

UPGMA and NMDS analysis indicated that epilithon differed significantly from neuston and plankton at the phylotype level, which was true for 16S rRNA, *pufM* and rhodopsin genes. There were “typical epilithon phylotypes” absent in water microbiomes and “typical water phylotypes” absent in epilithon. For instance, *Limnohabitans* and SAR11 (16S rRNA gene) were detected exclusively in neuston and plankton, whereas *Tabrizicola* and *Erythrobacter* (*pufM*) only in epilithon.

## Figures and Tables

**Table 1:** Physical and chemical characteristics of water samples. BK1, G1–surface microlayer; BK2, G2—water column; BK4, G4—bottom water. BK—samples taken off the Bol’shiye Koty settlement; G—samples taken off the Bol’shoye Goloustnoye settlement.

Sample	PO_4_^3−^, mg/L	NH_4_^+^, mg/L	NO_2_^−^, mg/L	NO_3_^−^, mg/L	N Total, mg/L	TOC, mg C/L	P Total, mg/L	T, °C
BK1	0.023	0.0125	0.0025	0.075	0.031	35.3	0.0225	13.7
BK2	0.008	0.002	0.001	0.08	0.022	5.8	0.007	10.0
BK4	0.016	0.004	0.001	0.29	0.073	5.4	0.008	6.0
G1	0.018	0.0085	0.0005	0.115	0.0315	3.1	0.015	15.4
G2	0.007	0.004	0.002	0.12	0.034	3.8	0.007	14.0
G4	0.009	0.007	0.002	0.17	0.046	3.7	0.007	12.0

**Table 2:** Chemical characteristics of biofilm samples. BK51, BK53—Bol’shiye Koty biofilms; G51, G52—Bol’shoye Goloustnoye biofilms.

Sample	N Total, mg/cm^2^	TOC, mg C/cm^2^	P Total, mg/cm^2^
BK51 + BK53	0.6	4.2	0.053
G51 + G52	0.4	2.7	0.024

**Table 3:** *pufM* gene sequences detected in Lake Baikal identified to the genus level.

ESV	Genus	Number of Seqs in Water Microbiomes	Number of Seqs in Epilithon	Closest Homologue Accession Number
278, 860, 1839, 2009	*Tabrizicola*	0	19507	QBQ34549
150, 2012, 2249	*Erythrobacter*	0	4033	AGK27892
2758	*Blastomonas*	4	1572	AGK27877
1430	*Blastomonas*	0	411	AGK27877
3609	*Sphingomonas*	0	855	AGK27878

**Table 4:** Alpha-diversity of *pufM*-containing microbial communities of epilithon (G51, G52, BK51, BK53), neuston (BK1, G1), and plankton (BK2, BK4, G2, G4) of Lake Baikal. BK—samples taken off the Bol’shiye Koty settlement; G—samples taken off the Bol’shoye Goloustnoye settlement.

Microbial Community	S.chao1	Shannon	Simpson
BK1	49	3.2	0.9
BK2	40	2.9	0.9
BK4	46	3.2	0.9
BK51	43	3.2	0.9
BK53	35	2.8	0.9
G1	47	3.1	0.9
G2	50	3.0	0.9
G4	34	2.7	0.9
G51	65	3.5	1.0
G52	52	3.0	0.9

**Table 5:** Rhodopsin gene sequences detected in Lake Baikal identified to the genus level.

ESV	Genus	Number of Seqs in Water Microbiomes	Number of Seqs in Epilithon	Closest Homologue Accession Number
1901, 2299	*Aquirufa*	821	0	WP_130895144
678	*Runella*	0	1330	TAG64876
1360	*Runella*	703	0	TAG64876

**Table 6:** Alpha-diversity of rhodopsin-containing microbial communities of epilithon (G51, G52, BK51, BK53), neuston (BK1, G1), and plankton (BK2, BK4, G2, G4) of Lake Baikal. BK—samples taken off the Bol’shiye Koty settlement; G—samples taken off the Bol’shoye Goloustnoye settlement.

Microbial Community	S.chao1	Shannon	Simpson
BK1	29	3.1	0.9
BK2	10	2.1	0.8
BK4	70	4.0	1.0
BK51	61	3.8	1.0
BK53	70	4.1	1.0
G1	133	4.6	1.0
G2	26	3.0	0.9
G4	26	2.9	0.9
G51	20	2.7	0.9
G52	46	3.5	1.0

## Data Availability

PRJNA613961 freshwater mixed marker sequence reads are available online. URL: https://dataview.ncbi.nlm.nih.gov/object/PRJNA613961?reviewer=6nqhjaovurg89onscgc6sn6br6 Last access date: 14 April 2021. Release date: 23 March 2024.

## Supplementary Materials

The following are available online at https://www.mdpi.com/article/10.3390/microorganisms9040842/s1, Figure S1: Rarefaction curves constructed from OTU_0.03_ and alpha-diversity of microbial communities of epilithon (G51, BK51, BK53), neuston (BK1, G1), and plankton (BK2, BK4, G2, G4) of Lake Baikal. BK–samples taken off the Bol’shiye Koty settlement; G–samples taken off the Bol’shoye Goloustnoye settlement, Table S1: PERMANOVA results for 16S rRNA samples based on Bray-Curtis distance matrix. ** P < 0.01, P < 0.1, Table S2: The average proportion of phyla detected in neuston, plankton, and epilithon of Lake Baikal according to 16S rRNA gene sequencing.
