# Supplementary material for: Diversity of Aerobic Anoxygenic Phototrophs and Rhodopsin-Containing Bacteria in the Surface Microlayer, Water Column and Epilithic Biofilms of Lake Baikal
Source: Microorganisms. 2021 Apr 14;9(4):842. doi: 10.3390/microorganisms9040842 (PMC8071047; doi:10.3390/microorganisms9040842)
Supplement: Supplementary file 1 [file microorganisms-09-00842-s001.zip › Table S1.docx]

**Table S1.** PERMANOVA results for 16S rRNA samples based on Bray-Curtis distance matrix. ** P < 0.01, . P < 0.1.

| **Variables** | **Df** | **SumOfSqs** | **R^2^** | **F** | **Pr(>F)** | **Significant** |
| --- | --- | --- | --- | --- | --- | --- |
| biotope | 1 | 0.896 | 0.456 | 6.924 | 0.003 | ** (P < 0.01) |
| station | 1 | 0.293 | 0.149 | 2.263 | 0.096 | . (P < 0.1) |
| Residual | 6 | 0.776 | 0.395 |  |  |  |
| Total | 8 | 1.964 | 1 |  |  |  |
