# Supplementary material for: Diversity of Aerobic Anoxygenic Phototrophs and Rhodopsin-Containing Bacteria in the Surface Microlayer, Water Column and Epilithic Biofilms of Lake Baikal
Source: Microorganisms. 2021 Apr 14;9(4):842. doi: 10.3390/microorganisms9040842 (PMC8071047; doi:10.3390/microorganisms9040842)
Supplement: Supplementary file 1 [file microorganisms-09-00842-s001.zip › Table S2.docx]

**Table S2.** The average proportion of phyla detected in neuston, plankton, and epilithon of Lake Baikal according to 16S rRNA gene sequencing.

| **Taxon** | **The share of sequences in**  **epilithon, %** | **The share of sequences in**  **neuston and plankton, %** |
| --- | --- | --- |
| Cyanobacteria | 17 | 23 |
| Actinobacteria | 22 | 19 |
| Firmicutes | 4 | 11 |
| Bacteroidetes | 4 | 10 |
| Proteobacteria | 34 | 24 |
| Fusobacteria | 3 | 6 |
| Verrucomicrobia | 9 | 5 |
| Acidobacteria | 4 | 0.9 |
| Chloroflexi | 1.8 | 0.5 |
| unclassified_bacteria | 0.03 | 0.4 |
| WPS-2 | 0.2 | 0.3 |
| Gemmatimonadetes | 0.1 | 0.3 |
| Armatimonadetes | 0.1 | 0.2 |
| Patescibacteria | 0.1 | 0.2 |
| FCPU426 | 0 | 0.1 |
| Planctomycetes | 0.7 | 0.1 |
| Nitrospirae | 0 | 0.05 |
| Dependentiae | 0.1 | 0 |
| Deinococcus-Thermus | 0.3 | 0 |
