# Supplementary figures and images for: Diversity of Aerobic Anoxygenic Phototrophs and Rhodopsin-Containing Bacteria in the Surface Microlayer, Water Column and Epilithic Biofilms of Lake Baikal
Source: Microorganisms. 2021 Apr 14;9(4):842. doi: 10.3390/microorganisms9040842 (PMC8071047; doi:10.3390/microorganisms9040842)

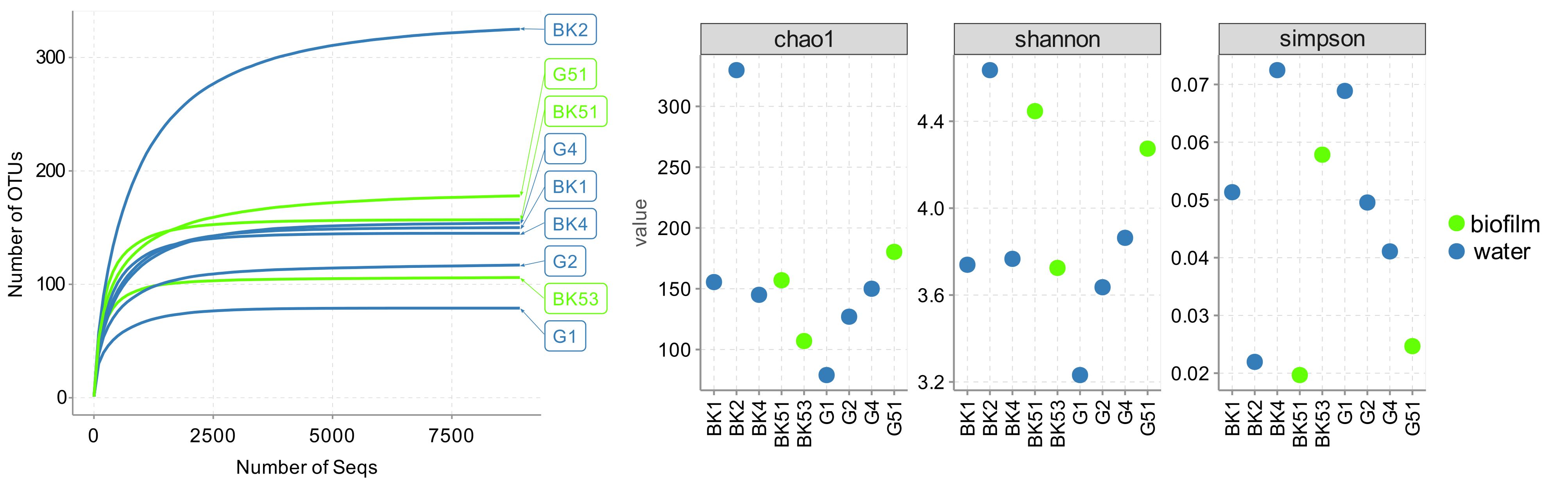

Supplement: Supplementary file 1 [file microorganisms-09-00842-s001.zip › FigS1.jpg]
